# Who Is Vulnerable to Dengue Fever? A Community Survey of the 2014 Outbreak in Guangzhou, China

**DOI:** 10.3390/ijerph13070712

**Published:** 2016-07-14

**Authors:** Bin Chen, Jun Yang, Lei Luo, Zhicong Yang, Qiyong Liu

**Affiliations:** 1 State Key Laboratory for Infectious Disease Prevention and Control, Collaborative Innovation Center for Diagnosis and Treatment of Infectious Diseases, National Institute for Communicable Disease Control and Prevention, Chinese Center for Disease Control and Prevention, Beijing 102206, China; drchenbin@126.com (B.C.); smart_yjun@163.com (J.Y.); 2 China CDC Key Laboratory of Surveillance and Early-Warning on Infectious Disease, Chinese Center for Disease Control and Prevention, Beijing 102211, China; 3 Xiamen Entry-Exit Inspection and Quarantine Bureau, Xiamen 361012, China; 4 Guangzhou Center for Disease Control and Prevention, Guangzhou 510440, China; llyeyq@163.com

**Keywords:** dengue fever, knowledge, lifestyle, risk factors, vector control

## Abstract

Unprecedented dengue fever (DF) outbreaks impel China to develop useful disease control strategies. Integrated vector management (IVM) focuses on identifying vulnerable populations and interrupting human–vector contact; however, vulnerable populations have not been clearly identified in China. We conducted a case-control study during the initial stage of the 2014 DF outbreak in Guangzhou, China to assess risk factors for DF infection. Cases were randomly sampled from the National Notifiable Infectious Disease Reporting Information System (NNIDRIS). Controls were healthy individuals recruited from 17 DF infected communities through cluster sampling. A structured questionnaire on demographics, knowledge, practices, and living environment was administered to participants (165 cases; 492 controls). Logistic regression models identified characteristics of vulnerable populations. Awareness of dengue (OR = 0.08, 95% CI = 0.04–0.17), removing trash and stagnant water from around the residence (OR = 0.02, 95% CI = 0.00–0.17), and using mosquito repellent oils (OR = 0.36, 95% CI = 0.16–0.81) were protective factors. Living in an old flat or shed (OR = 2.38, 95% CI = 1.18–4.79) was a risk factor. Coils and bed nets were not protective due to incorrect knowledge of use. Using mosquito repellent oils and other protective measures can reduce vulnerability to DF infection.

## 1. Introduction

Dengue fever (DF) is the most prevalent mosquito-borne viral disease in humans, and has spread rapidly within and across countries over the past few decades [1]. It is also an emerging neglected tropical disease (NTD) with geographical distribution in South China [2]. From 1978 to 2008, a total of 655,324 cases were reported in Mainland China, resulting in 610 deaths [3]; from 2009 to 2014, a total of 52,749 DF cases and 6 deaths were reported for the same land area [4]. Ooi notes that the increasing number of reports of DF outbreaks in various parts of South China is likely attributable to multiple factors, most significantly the increase in trade and human movement through China from the Southeast Asian region where DF is firmly endemic [5]. In addition, Guangzhou and other cities in South China are growing economically, leading to continuous expansion of city size and population density. In Guangzhou, the capital city of Guangdong province in South China has a population of 12.9 million and a population density of 1739 per km^2^ [6]. Instead of merely importing DF, South China could become an epicenter of DF transmission to other parts of China, and possibly the rest of the world, if proper preventive measures are not taken.

Although the Chinese government has taken surveillance and control measures for DF, such as strengthening surveillance and conducting risk communication with the public [7], the total number of dengue fever cases reached 41,155 in Guangdong in 2014. More than 80% of cases were reported in Guangzhou City and its neighboring prefectures [8]; the horrendous outbreak in Guangzhou lead to 37,359 cases and 5 deaths. Incidence soared to greater than 290 per 100,000 population, making it the most severe DF outbreak since the 1980s. It appeared that current DF prevention strategies and vector control programs were inadequate [9]. 

Preventing DF virus transmission depends entirely on controlling the mosquito vectors and interrupting human–vector contact. In 2004, the World Health Organization (WHO) adopted the Global Strategic Framework on Integrated Vector Management (IVM) as a first step towards implementation of a new approach to vector control [10]. The IVM strategy is an integrated disease management approach that incorporates all components of disease control, including vector control, prevention, treatment, and reducing human vulnerability [11]. However, fundamental factors contributing to DF transmission need to be identified, e.g., it is necessary to determine people who are vulnerable to DF transmission in South China. Previous research of a Caribbean population reports that the most vulnerable population is the poor, because water storage is critical for their daily life and adequate knowledge of DF is often lacking [12]. In addition, studies in Malaysia, Pakistan, and Thailand have linked socioeconomic and behavioral factors; and community knowledge, attitudes, and practices with DF infection rates [13,14,15,16,17,18,19]. To the best of our knowledge, similar studies have not been conducted in China. *Aedes albopictus* is the sole vector for dengue transmission in Guangzhou [20], and no published reports have detailed community vulnerability to *Aedes albopictus* transmission of DF in the epidemic season. In addition, there is limited information on how vulnerable populations of China respond to DF outbreaks and what preventive measures are taken. In order to implement effective disease control strategies for DF in South China, we used data from the 2014 DF outbreak investigation in Guangzhou, China to analyze population vulnerability and identify the main risk factors for DF transmission.

## 2. Materials and Methods 

### 2.1. Data Collection

We performed a community-based case-control study. Data were collected on human exposure to mosquitoes, living environment, knowledge of DF, and preventive behaviors for vector control from the participants.

### 2.2. Cases

Cases were randomly sampled from the National Notifiable Infectious Disease Reporting Information System (NNIDRIS), a web-based reporting system for Mainland China [21]. In China, DF is a notifiable disease and all cases of DF are diagnosed according to the unified diagnostic criteria issued by Chinese National Health and Family Planning Commission, which includes definitions of clinically diagnosed and laboratory confirmed cases [22]. Registered clinically diagnosed cases were identified by experienced local physicians according to clinical manifestations and exposure history. Laboratory confirmed cases were determined based on clinically diagnosed cases presenting with any of the following lab test results relating to DF: a 4-fold increase in specific IgG antibody titer, positive PCR test, or positive virus isolation and identification test. Both clinically diagnosed and laboratory confirmed cases were included in this study. All cases were reported in NNIDRIS between 30 June and 30 August 2014. 

We performed a random sample telephone interview of cases with a registered telephone number in the NNIDRIS using a random number table. Attempts were made to call the numbers between 1 September and 10 September 2014. Tele-interviewers who could speak Mandarin and Cantonese were recruited from Southern Medical University and Guangzhou Medical University. Interviews were conducted between 17:30 and 22:00 on weekdays, in order to avoid over-representation of unemployed participants, and from 10:00 to 19:00 on weekends or public holidays. During each call, after the study was explained and verbal consent was obtained, the respondent was asked to complete the survey. If the respondent was busy, a call was made later when the respondent was available to finish the questionnaire. Interviewers made three attempts to call unanswered telephones on different days before categorizing them as “did not respond.” All completed tele-interview questionnaires were reviewed by a public health practitioner at a community health center in Guangzhou to ensure their validity.

### 2.3. Controls 

An eligible community control was defined as a healthy, uninfected individual who resided in a community or neighborhood infected with DF. Controls were chosen from newly DF infected neighborhoods to eliminate the influence of previous epidemics but still match the living environment of DF cases. Control groups were selected using a multistage simple random sampling procedure, with a cluster sampling procedure as the final stage. In the first stage, 45 communities newly affected by the DF epidemic from 10 August to 24 August 2014 were listed. To obtain an appropriate total sample, 17 communities were randomly selected for the control group. In the second stage, a list of all households from the 17 communities was made, from which 30 households within a 400 m radius of newly confirmed diagnosis DF patients were randomly selected. In the third stage, all household members in the selected households aged 12 years and older were selected and invited for an interview. In the fourth and final stage, we randomly chose one person from each selected household for an interview. History of DF infection was checked through self-reported history of DF infection and examining registered Identification Numbers from NNIDRIS. If a selected individual had experienced suspected symptoms of DF in the past three months or was diagnosed with DF, they were deleted from the database. To improve cooperation, each interviewee received a small gift worth 18 Chinese Renminbi (6.2 Renminbi = $1 US), such as a bottle of mosquito repellent oil, after the survey was completed. All selected participants provided informed consent to be interviewed. Average survey completion time was 10–15 min. 

### 2.4. Study Sample 

Sample size was calculated using Epi Info™ StatCalc (Version 7, Centers for Disease Control and Prevention, Atlanta, GA, USA). The following assumptions were made during the calculation: 95% confidence interval, 80% power, and 10% wasting in controls. The minimum odds ratio (OR) for the association between cases and controls was set at 2.6, based on a pre-interview in Guangzhou city and a previous report that air conditioner absence was significantly associated with DF Immunoglobulin M (IgM) seropositivity (OR = 2.6) [23]. The ratio of cases to controls was set at 1:3. Adding a 20% non-response rate into these assumptions led to an estimated required sample size of 604 (151 cases and 453 controls). In order to address potentially low response rates in the tele-interview, we selected 10% of all registered DF cases to reach the sample size for the case group.

### 2.5. Survey Instruments

All surveys were conducted using the same questionnaire, which was pretested for face and content validity, length, and comprehensibility. We investigated socio-demographic characteristics, building structures and surrounding environment of the household, information on mosquito exposure, self-reported preventive practices against DF infection, and knowledge of DF. Cases were asked to recall their knowledge and behaviors before their illness.

### 2.6. Data Analysis

Data were double entered and crosschecked using EpiData (Version 3.1, The EpiDataAssociation, Odense, Denmark). The variables used in this study were analyzed using bivariate and multivariate methods. Crude associations between DF infection and vulnerability variables were analyzed using chi-square tests, *t*-tests, and Wilcoxon Rank-Sum tests. Significant risk factors were selected through stepwise regression using backward elimination to determine a final multivariate logistic regression model. The significance of risk factors was estimated using standard partial regression coefficients and ORs. The logistic regression model used DF infection as the dependent variable and 11 independent variables as possible covariates (i.e., occupation, severe mosquito bites, residence in old flats, lacking an air conditioner, plants with water containers, awareness of DF, using repellent oils as a preventive measure, using net coils, using bed nets, using screens for windows and doors, and cleaning trash and water out of the dwelling). The input *p*-value of variables was 0.05 and the output *p*-value of variables was 0.10. The goodness-of-fit of the logistic regression models was tested by the summary measures based on the likelihood chi-square statistics. All statistical analyses were performed with IBM SPSS Statistics 20.0 (IBM, Chicago, IL, USA). In all analyses, a *p*-value < 0.05 was considered statistically significant. 

### 2.7. Ethical Consideration

Ethical approval for this project was obtained by the Chinese Center for Disease Control and Prevention Ethical Review Committee (No. 201214). Patient data used in the study were de-identified. Respondents were assured that their responses would remain confidential and anonymous, and that they were free to withdraw from the interview at any time. As written informed consent is not practical in a telephone survey, verbal informed consent was obtained from the respondents prior to each interview. The verbal consent procedure was approved by the Medical Ethics Committee.

## 3. Results

### 3.1. Demographic Characteristic

**Figure 1:**
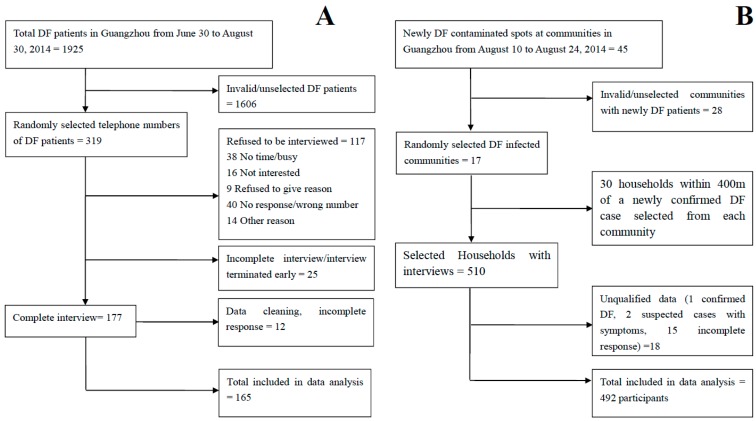
Flow charts for the recruitment of cases and controls to determine populations vulnerable to Dengue Fever infection in the 2014 Guangzhou outbreak. (**A**) Flowchart for telephone surveys conducted with dengue fever (DF) cases; (**B**) Flowchart for face-to-face interviews of controls conducted at 17 local communities.

A total of 319 cases were successfully contacted; 177 cases responded to the survey, of which 165 completed the full questionnaire (Figure 1A). The majority (81.1%) of cases were diagnosed by local experienced physicians according to clinical manifestations and exposure history; the remaining 18.9% were diagnosed through laboratory confirmation. Among the interviews, 85 cases (47.8%) were ever hospitalized for DF treatment. 

We were unable to contact all cases for various reasons including: calls not being answered, phone number was a “wrong number”, the telephone number was in use by others during the interview, and the service was terminated for the telephone number. The most common reasons used by participants when refusing to take part in the survey were: “Too busy”, “It is boring”, and “Not interested”. The data were edited to identify and remove incomplete responses, leaving a total of 165 complete responses for analysis. The response rate was 51.7% (computed as the number of completed responses divided by the number of eligible and contacted patients; Figure 1A). There were 510 households selected for interviews. Data from eighteen households were deleted. We identified one confirmed DF case and two suspected cases with symptoms; the remaining provided incomplete responses. Figure 1B outlines selection of the 492 community controls.

The demographic features of participants are summarized in Table 1. There were no significant differences in age, gender distribution, and migrant status between cases and controls. The mean age of cases was 37.87 ± 14.93 years old (median: 37 years old; range: 12 to 69 years old) compared to 39.20 ± 15.01 years old (median: 36 years old; range: 13 to 70 years old) for controls. There were significant differences in proportions of occupations between case and control groups; the majority of DF cases were merchants (19.9%), office workers (17.1%), and retired persons (16.4%), compared to unemployed (25.2%), retirees (21.8%), and office workers (21.4%) for community controls.

### 3.2. Univariate Analysis

#### 3.2.1. Exposure Characteristics

**Figure 2:**
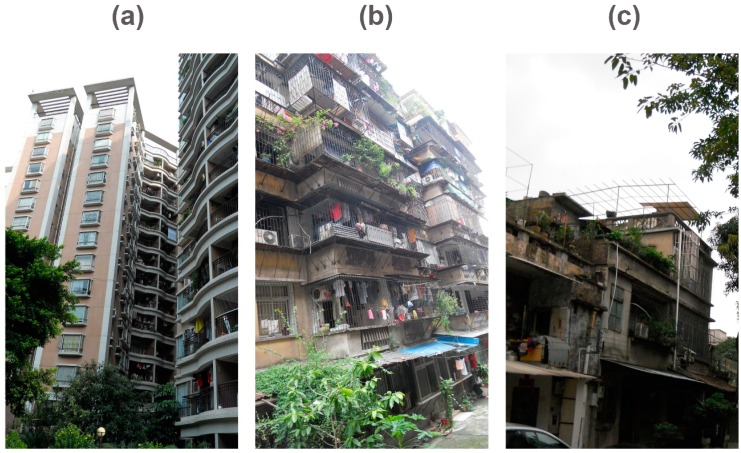
Types of residential buildings in the 2014 Guangzhou outbreak (from left to right: (**a**) high-rise modern apartment; (**b**) old flat; (**c**) shed).

The proportion reporting severe mosquito bites was significantly higher in DF cases than controls (51.2% vs. 38.9%, χ^2^ = 7.24; *p* = 0.007) (Table 1). Respondents reported three main types of living environment: high-rise modern apartments (equipped with elevators), old flats, and sheds (Figure 2). The proportion of DF cases living in old flats and sheds was higher than controls (80.1% vs. 63.0%, χ^2^ = 29.411; *p* < 0.001). In addition, 56% of DF cases lived without an air conditioner or only occasionally used one, compared to 38% of controls (χ^2^ = 9.578; *p* = 0.002). Few respondents reported having a household equipped with screened windows or doors, and the proportion was higher in controls than in cases (28.30% vs. 18.20%, χ^2^ = 6.318; *p* = 0.012). Controls were more likely to have plants with water containers compared to cases (40.40% vs. 18.60%, χ^2^ = 24.317; *p* < 0.001); however, controls more frequently changed water in the plant containers than cases, although this difference was not statistically significant (58.3% vs. 35.3%, χ^2^ = 3.303; *p* = 0.069).

#### 3.2.2. Knowledge and Practices

Almost two-thirds (60.0%) of DF cases had DF knowledge, before they became infected; this proportion was significantly lower than that in controls (χ^2^ = 19.09, *p* < 0.001) (Table 1). This study also found lower proportions of mosquito repellent oil use as a preventive measure in cases than controls (24.2% vs. 34.1%, χ^2^ = 5.272; *p* = 0.022). Moreover, fewer cases removed trash and water from around their residence compared to controls (9.1% vs. 28.5%, χ^2^ = 25.081; *p* < 0.001). The proportions of mosquito coil and bed net use were significantly higher in cases than controls. There were no significant differences between the two groups in terms of spraying insecticide within their homes or wearing long-sleeved shirts and long pants. 

### 3.3. Multivariate Analysis

Of the covariates assessed, awareness of dengue, removing trash and standing water from around the residence, living in an old flat or shed, using mosquito coils, using bed nets, using mosquito repellent oils, and having plants with water containers had significant associations with DF infection (Table 2). Among them, awareness of dengue (OR = 0.08, 95% CI = 0.04–0.17), removing trash and standing water from around the residence (OR = 0.02, 95% CI = 0.00–0.17), using mosquito repellent oils (OR = 0.36, 95% CI = 0.16–0.81), and planting with water containers (OR = 0.43, 95% CI = 0.21–0.87) were found to be protective factors. Living in old flats or sheds were found be risk factors (reference = high-rise modern apartment; OR = 2.38, 95% CI = 1.18–4.79). The use of mosquito coils (OR = 2.812, 95% CI = 1.508–5.244) and bed nets (OR = 2.921, 95% CI = 1.574–5.422) were risk factors for DF infection (Table 2). 

## 4. Discussion

Several studies have examined risk factors for DF transmission; however, few studies have examined the population vulnerability factors important for DF vector control in South China [13,17,24,25,26]. Our study detailed individual knowledge and lifestyle features of populations vulnerable to DF, which will help to guide effective community interventions in the future.

Importantly, DF awareness, removing trash and water from around the dwelling, and using mosquito repellent oils were associated with decreased risk of DF infection. A national survey of the Malaysian public reported that higher dengue knowledge results in better dengue prevention practices [25], indicating that a lack of knowledge of dengue transmission leads to vulnerability. As detected in our study, the rate of recognizing DF in cases was lower than controls. Furthermore, a multi-country study showed that the negative association between dengue vectors and individual knowledge was mediated by behavior change [27]. Our findings presented evidence that clearing trash and standing water from around the dwelling were protective practices; this finding is supported by a report from Thailand that showed a significant reduction of dengue vectors and DF cases in areas that had cleanup campaigns before and during rainy seasons [18]. Previous research indicates that indoor ornamental plants are associated with DF infection, and stagnant water in the pots of plants are common breeding grounds for *Aedes* mosquitoes in high-rise buildings in Malaysia [19,28,29]. Unexpectedly, we found that indoor plants with water containers were not a risk factor for DF transmission, and controls were more likely to plant with water containers than cases (40.40% vs. 18.60%). One explanation is that controls in Guangzhou changed the water in plant containers more frequently than cases. Another explanation is that rate of indoor air conditioner use was higher among controls than cases. A study of DF that examined the Texas-Mexico border showed that even though *Aedes aegypti* were more abundant in Texas, the incidence of DF cases in Texas was lower than in Nuevo Laredo, Mexico due to indoor air conditioner use [23].

An additional risk factor for DF infection was living in old flats or sheds (i.e., temporary buildings). Most DF cases (80.1%) in Guangzhou lived in old flats without an elevator, which are often cramped and have poor sanitation. This frequency of cases residing in poor living conditions is consistent with previous research in Vietnam [24]. Guangzhou has a subtropical monsoon climate with hot and humid summers, which leads to a high prevalence of air conditioners in households. However, we found that more than half (56%) of DF cases lived without an air conditioner or only occasionally used one, compared to only 38% of controls. In addition, few households were equipped with screened windows or doors, which could contribute to DF infection vulnerability. Modern housing, air conditioners, and screens are often not available to those of low socioeconomic status. An investigation of urban DF found that differences in transmission rates were attributable to neither climate nor vector populations, but instead to socioeconomic factors such as air conditioner use [23]. It is predicted that air conditioner use may become even more prevalent if climate change continues to increase temperatures worldwide, which could potentially reduce DF transmission by decreasing time spent outdoors and reducing exposure to vectors that enter homes through open windows. However, the ability to afford an air conditioner would still be dependent on socioeconomic status.

Surprisingly, using mosquito coils and bed nets were not protective factors for DF transmission in Guangzhou. Conversely, they were found to be risk factors. We identified that most residents chose mosquito coils and bed nets as preventive measures while sleeping, which turned out to have limited impact on *Aedes albopictus* bites. *Aedes albopictus* has a strong preference for human feeding and biting with a bimodal activity peak, typically around two hours before sunset (the largest peak of activity) and 8:30 a.m. (a smaller peak) [30]. Thus, night activity for *Aedes albopictus* is minimal or absent, as confirmed by research in New Delhi, India [30,31]. In addition, we also found that severe mosquito bites were significantly higher among cases than controls, which may have led to the choice to use coils and bed nets as preventive measures. Hence, precise knowledge and effective measures should be appropriately translated to public. Strengthening the public’s capacity for adaptive measures and awareness, especially accurately recognizing and understanding DF vectors, should be included in the outbreak response.

Lai and colleagues warned that the possibility exists that the receptivity and vulnerability of certain areas to DF outbreaks could be increasing, highlighting that urgent steps need to be taken [32]. Various factors may influence a community’s vulnerability to dengue epidemics; the distribution and density of the human population, settlement characteristics, conditions of land tenure, housing styles, education, and socio-economic status are all interrelated and fundamentally important for planning and assessing the risk of DF outbreaks [33]. Through our analysis, we concluded that populations living in old flats or sheds, with poor or incorrect knowledge of DF transmission factors, and reluctant to clear trash and standing water from around the residence were more vulnerable to DF infection. Thus, novel DF interventions should take into account these target populations to increase adoption of DF preventive behaviors in order to reduce transmission and future outbreaks [14]. WHO promotes IVM to improve urban infrastructure and basic services for vulnerable populations and to reduce available larval habitats and vector contact [34]. All in all, we determined vulnerable populations and the main risk factors important for controlling dengue virus transmission in Guangzhou. Further studies should identify other protective behaviors such as health seeking behaviors and additional socio-economic factors for population vulnerabilities, and find adaptive approaches to implement targeted interventions. 

An important strength of this case-control study is that it takes into account recently diagnosed DF cases, and chose healthy populations in newly DF infected communities as controls at the initial stage of a DF outbreak. This method avoided bias from the influence of other surveys or interventions that would have already been implemented in endemic areas. We also screened variables of interest through backward step-wise logistic regression, which identified significant predictors of DF infection and allowed us to draw conclusions on lifestyle factors and knowledge associated with acquiring DF. 

Several limitations of this study need to be mentioned. One limitation was the use of self-reported data on some preventive measures using a questionnaire, which is, by definition, subjective. The effect of this form of bias was reduced by asking cases and controls the same questions. Another limitation was that telephone surveys of cases only included households with working telephones, even though every case had detailed contact information; therefore, individuals without telephone lines, such as those who are socioeconomically disadvantaged, were underrepresented. We put forth best efforts to interview their families and check other information through public health practitioners at the community health center in Guangzhou. The third limitation was control group recruitment. We could not perform blood tests to identify uninfected individuals. Hence, we inquired about disease symptoms of participants and checked that they were not reported in the NNIDRIS. Our research only focused on identifying populations vulnerable to DF infection to provide evidence for novel prevention and control strategies. However, future studies should address this topic with serological investigation to further reduce potential bias. Lastly, recall bias may exist in the present study, though we chose newly diagnosed DF patients and investigated them through telephone calls in time to reduce bias.

## 5. Conclusions 

Our findings demonstrate five main population vulnerabilities to DF infection: living in old flats, poor or incorrect knowledge of DF, lower use of preventive practices such as removing trash and standing water from around the residence, and lower use of mosquito repellent oil. It is evident that there need to be more targeted interventions to improve adaptive capacity among populations vulnerable to DF. Future studies should further analyze the influence of factors leading to DF vulnerability, and focus on effective protective measures. 

## Figures and Tables

**Table 1:** Univariate analysis of selected variables for Dengue in Guangzhou outbreak 2014, China.

Variable		Controls No. (%) (*n* = 492)	Cases No. (%) (*n* = 165)	*p*-Value
Gender (male)		222 (45.4)	73 (44.2)	0.796
Age (year)	Mean (SD)	39.20 (15.01)	37.87 (14.93)	0.643
	Median (Min.–Max.)	36 (13–70)	37 (12–69)	0.367
Migrant person		181 (36.8)	54 (32.7)	0.346
Occupation				<0.001
	Farmer	15 (3.2)	8 (5.5)	
	Merchant	62 (13.2)	29 (19.9)	
	Office worker	100 (21.4)	25 (17.1)	
	Laborer	22 (4.7)	23 (15.8)	
	Unemployed	118 (25.2)	20 (13.7)	
	Retiree	102 (21.8)	24 (16.4)	
	Pupils/student	49 (10.5)	17 (11.6)	
Severe mosquito bites		157 (38.9)	83 (51.2)	0.007
Living in old flats/sheds		308 (63.0)	129 (80.1)	<0.001
Lacking air conditioner		187 (38.0)	47 (56.0)	0.002
Plants with water containers				
	Lucky bamboo plant	163 (40.4)	30 (18.6)	0.000
	Frequently change water	95 (58.3)	6 (35.3)	0.069
Awareness of Dengue		381 (77.4)	99 (60.0)	0.000
Preventive Measures				
	Repellent	137 (34.1)	40 (24.2)	0.022
	Coil	140 (34.7)	101 (61.2)	<0.001
	Net	126 (31.3)	88 (53.3)	<0.001
	Screen	114 (28.3)	30 (18.2)	0.012
	Spray	32 (7.9)	15 (9.1)	0.651
	Clothes	13 (3.2)	7 (4.2)	0.554
	Cleaning trash/water	115 (28.5)	15 (9.1)	<0.001

**Table 2:** Logistic regression model results for predictors of Dengue Fever infection in the 2014 Guangzhou outbreak.

Variables	*B*	S.E	*p* Value	Odds Ratio(OR)	95% CI
Awareness of Dengue	−2.538	0.401	<0.001	0.08	0.04–0.17
Living in old apartment	0.866	0.358	0.015	2.38	1.18–4.79
Plants with water containers	−0.853	0.363	0.019	0.43	0.21–0.87
Preventive Measures					
Repellent	−1.033	0.418	0.013	0.36	0.16–0.81
Nets	1.069	0.322	0.001	2.91	1.55–5.48
Cleaning trash/water	−3.901	1.101	<0.001	0.02	0.00–0.17
Coils	0.978	0.318	0.002	2.66	1.43–4.96
Constant intercept	−0.285	0.435	0.512	0.75	

## Supplementary Materials

The following are available online at www.mdpi.com/1660-4601/13/7/712/s1, Text S1: The summary of Diagnostic Criteria for Dengue Fever by Chinese Ministry of Health (WS 216-2008), Table S1: The specification of variable assignment, Table S2: Variables not in the Equation of logistic regression.
