# Supplementary material for: Who Is Vulnerable to Dengue Fever? A Community Survey of the 2014 Outbreak in Guangzhou, China
Source: Int J Environ Res Public Health. 2016 Jul 14;13(7):712. doi: 10.3390/ijerph13070712 (PMC4962253; doi:10.3390/ijerph13070712)
Supplement: Supplementary file 1 [file ijerph-13-00712-s001.pdf]

Who Is Vulnerable to Dengue Fever? A Community Survey of the 2014 Outbreak in Guangzhou, China

Bin Chen, Jun Yang, Lei Luo, Zhicong Yang and Qiyong Liu

Text S1. The summary of Diagnostic Criteria for Dengue Fever by Chinese Ministry of Health (WS 216-2008). (Issued on 28 February 2008)

1. Epidemiologic Linkage

1.1. Travel to a dengue endemic country/region within previous 14 days of dengue-like illness
1.2. Around the place of residence or place of work (e.g., 100 m radius), there have been dengue case(s) within one month.

2. Clinical Description

2.1. Sudden onset, fever (39–40 °C within 24–36 h, someone shows biphasic fever); severe headache, retro-orbital pain, myalgia, arthralgia and fatigue; flushed skin on face, neck and chest, and conjunctival congestion, etc.
2.2. Rash: measles-like rash, scarlatiniform rash, and/or needle-like hemorrhagic rash in the limbs, trunk, head and face in the course of illness (days 5–7); itching; no scaling; continued 3–5 days.
2.3. Bleeding tendency (tourniquet test positive): petechia, ecchymoses, purpura and injection site bleeding, or bleeding from the mucous membranes of mouth and nose, gastrointestinal bleeding, hemoptysis.
2.4. Hematuria and vaginal bleeding in the course of illness (days 5–8).
2.5. Massive hemorrhage of gastrointestinal tract, or chest and abdominal cavity bleeding, or intracranial hemorrhage.
2.6. Liver enlargement, pleural or pericardial effusion.
2.7. Shock syndrome: clammy skin, restlessness, rapid and weak pulse and narrow pulse pressure < 20 mmHg (2.7 kPa) and undetectable in blood pressure, oliguria etc.

3. Laboratory Tests

3.1. A total white blood cell count decrease.
3.2. Thrombocytopenia ($<100 \times 10^9$/L).
3.3. Hemoconcentration (an increase in hematocrit ≥20% above average for age or a decrease in hematocrit ≥20% of baseline following fluid replacement therapy); hypoproteinemia.
3.4. IgG or IgM anti-DENV positive in a serum specimen.
3.5. Cell culture isolation of DENV by *Aedes albopictus* C6/36 cell or 1–3 day-old newborn mice in acute serum, cerebrospinal fluid, blood, or other tissue specimens.
3.6. IgG anti-DENV ≥ 4-fold rise in titer in paired acute and convalescent serum samples. The serologic tests included ELISA, mac-ELISA, HI, FA/IFA, NT.
3.7. Detection of DENV nucleic acid by RT-PCR or real-time fluorescence quantitative PCR.

4. Diagnosis and Classification

4.1. Suspected case: a patient with item 1.1 and 2.1, or a patient with item 2.1, 3.1 and 3.2, as defined above.
4.2. Clinically diagnosed case:

DF: a suspected case with 1.2, 3.1 and 3.2; or a suspect case with item 2.1, 3.1, 3.2 and 3.4
DHF: a clinically diagnosed case of DF with item 3.2, 3.3 and one of item 2.3 to 2.5.
DSS: a clinically diagnosed case of DHF with item 2.6.

4.3. Confirmed case: a clinically diagnosed case with oneof item 3.5 to 3.7.

Table S1. The specification of variable assignment.

Variables	Specification	Remarks
Occupation	1 = Farmer, 2 = Merchant, 3 = Office worker, 4 = Laborer, 5 = Unemployed, 6 = Retiree, 7 = Student (as reference of dummy variables)	Covariates Variable
Using repellent	0 = No, 1 = Yes	Covariates Variable
Using bed net	0 = No, 1 = Yes	Covariates Variable
Cleaning trash/water of dwellings	0 = No, 1 = Yes	Covariates Variable
Screen windows and doors	0 = No, 1 = Yes	Covariates Variable
Using mosquito coils	0 = No, 1 = Yes	Covariates Variable
Plant with water container	0 = No, 1 = Yes	Covariates Variable
Awareness of Dengue	0 = No, 1 = Yes	Covariates Variable
mosquito bites	0 = mild, 1 = severity	Covariates Variable
Using air-conditioner	1 = Lacking air conditioner (or using it less than six hours), 0 = Using air-conditioner regularly (or using it more than six hours)	Covariates Variable
Type of housing	1 = Living in old apartment/sheds/temporary building, 0 = High-rise building (with elevator)	Covariates Variable
Dengue fever	0 = Negative, 1 = Positive	Dependent Variable

Table S2. Variables not in the Equation of logistic regression.

Steps	Variables Evaluation	Excluded Variables	Score	df	Sig.
Step 2 [a]	Variables	Screen	0.104	1	0.747
	Overall Statistics		0.104	1	0.747
Step 3 [b]	Variables	Mosquito bites	1.004	1	0.316
		Screen	0.028	1	0.866
	Overall Statistics		1.106	2	0.575
Step 4 [c]	Variables	Occupation (student as reference)	7.115	6	0.31
		Occupation (Farmer)	1.658	1	0.198
		Occupation (Merchant)	3.148	1	0.076
		Occupation (Office worker)	0.163	1	0.687
		Occupation (Laborer)	0.706	1	0.401
		Occupation (unemployed)	2.422	1	0.12
		Occupation (Retiree)	0.058	1	0.81
		Mosquito bites	0.883	1	0.347
		Screen	0.023	1	0.878
	Overall Statistics		8.208	8	0.413
Step 5 [d]	Variables	Lack of air-conditioner	2.158	1	0.142
		Occupation (student as reference)	7.024	6	0.319
		Occupation (Farmer)	1.211	1	0.271
		Occupation (Merchant)	2.893	1	0.089
		Occupation (Office worker)	0.309	1	0.578
		Occupation (Laborer)	1.213	1	0.271
		Occupation (unemployed)	2.411	1	0.12
		Occupation (Retiree)	0.126	1	0.723
		Mosquito bites	0.875	1	0.35
		Screen	0.027	1	0.87
	Overall Statistics		10.317	9	0.325

[a] Variable(s) removed on step 2: Screen; [b] Variable(s) removed on step 3: Mosquito bites; [c] Variable(s) removed on step 4: Occupation; [d] Variable(s) removed on step 5: Lack of air-conditioner.

© 2016 by the authors; licensee MDPI, Basel, Switzerland. This article is an open access article distributed under the terms and conditions of the Creative Commons by Attribution (CC-BY) license (http://creativecommons.org/licenses/by/4.0/).
